# Clinical Efficacy Protocol of Yinhuapinggan Granules: A Randomized, Double-Blind, Parallel, and Controlled Clinical Trial Program for the Intervention of Community-Acquired Drug-Resistant Bacterial Pneumonia as a Complementary Therapy

**DOI:** 10.3389/fphar.2022.852604

**Published:** 2022-06-30

**Authors:** Jiaoli Wang, Haoran Hu, Haixia Du, Man Luo, Yilan Cao, Jiaping Xu, Tianhang Chen, Yilei Guo, Qixiang Li, Wen Chen, Yifei Zhang, Jin Han, Haitong Wan

**Affiliations:** ^1^ Zhejiang Chinese Medical University, Hangzhou, China; ^2^ Department of Respiratory Medicine, Hangzhou First People’s Hospital, Hangzhou, China; ^3^ College of Basic Medical Science, Zhejiang Chinese Medical University, Hangzhou, China; ^4^ College of Life Science, Zhejiang Chinese Medical University, Hangzhou, China; ^5^ Dongzhimen Hospital, Beijing University of Chinese Medicine, Beijing, China

**Keywords:** Yinhuapinggan (YHPG, ), traditional Chinese medicine (TCM), community-acquired drug-resistant bacterial pneumonia (CDBP), multidrug resistance, clinical trial, clinical efficacy

## Abstract

**Background:** Community-acquired bacterial pneumonia (CABP) is an important health care concern in the worldwide, and is associated with significant morbidity, mortality, and health care expenditure. *Streptococcus pneumoniae* is the most frequent causative pathogen of CABP. Common treatment for hospitalized patients with CABP is empiric antibiotic therapy using β-lactams in combination with macrolides, respiratory fluoroquinolones, or tetracyclines. However, overuse of antibiotics has led to an increased incidence of drug-resistant S. *pneumoniae*, exacerbating the development of community-acquired drug-resistant bacterial pneumonia (CDBP) and providing a challenge for physicians to choose empirical antimicrobial therapy.

**Methods:** Traditional Chinese medicine (TCM) is widely used as a complementary treatment for CDBP. *Yinhuapinggan* granules (YHPG) is widely used in the adjuvant treatment of CDBP. Experimental studies and small sample clinical trials have shown that YHPG can effectively reduce the symptoms of CDBP. However, there is a lack of high-quality clinical evidence for the role of YHPG as a complementary drug in the treatment of CDBP. Here, we designed a randomized, double-blind, placebo-controlled clinical trial to explore the efficacy and safety of YHPG. A total of 240 participants will be randomly assigned to the YHPG or placebo group in a 1:1 ratio. YHPG and placebo will be added to standard treatment for 10 days, followed by 56 days of follow-up. The primary outcome is the cure rate of pneumonia, and the secondary outcomes includes conversion rate of severe pneumonia, lower respiratory tract bacterial clearance, lactic acid (LC) clearance rate, temperature, C-reactive protein (CRP), criticality score (SMART-COP score), acute physiological and chronic health assessment system (APACHEII score) and clinical endpoint events. Adverse events will be monitored throughout the trial. Data will be analyzed according to a pre-defined statistical analysis plan. This research will disclose the efficacy of YHPG in acquired drug-resistant pneumonia.

**Clinical Trial Registration**: https://clinicaltrials.gov, identifier ChiCTR2100047501

## Introduction

Community-acquired bacterial pneumonia (CABP) is a frequent clinical condition and one of the most common infectious diseases (Sheam et al., 2020). Commonly used treatments include anti-infective therapy, general therapy and symptomatic treatment, of which antibiotic medication is the most important. However, as a double-edged sword, antimicrobial drugs are also an important cause of community-acquired bacterial drug-resistant pneumonia (CDBP) (Kollef and Betthauser, 2019; Wongsurakiat and Chitwarakorn, 2019). With the increasing use of antibiotics worldwide (Klein et al., 2018), the crisis of drug resistance has become more and more serious, and has been listed as a major public health problem by the World Health Organization (Emily et al., 2011). Management of CDBP has been further complicated due to the emergence of antibiotic resistance, particularly from *Streptococcus pneumoniae* strains (Liapikou et al., 2019). Published guidelines for the management of CDBP provide health care practitioners with current, evidence-based standards for antimicrobial therapy, yet even this expert guidance sometimes conflicts (van Duin and Paterson, 2016; Cao et al., 2018; Froes et al., 2019; Torres et al., 2019). Inflammatory diseases of the respiratory system caused by drug-resistant bacterial infections are commonly in respiratory, emergency and intensive care wards. Recently there has been an increasing number of highly hypervirulent carbapenem-resistant *K. pneumoniae* infections (Nair and Niederman, 2021), which might cause blood stream infection or nosocomial pneumonia related sepsis (Lee et al., 2017). As the drug resistance of bacteria makes antibacterial drugs less effective, it is difficult to suppress the expansion of inflammatory cells, which in turn leads to increased lung damage. If the inflammation spreads further, it can cause serious conditions such as waterfall uncontrolled systemic inflammatory response and multi-organ failures, where the morbidity and mortality rate are extremely high (He et al., 2018).

Recent studies, including multiple randomized controlled trials and systematic reviews, have demonstrated that short-term antibiotic therapy is safe and equally effective for most patients with pneumonia (Avdic et al., 2012; Haas et al., 2016; Li et al., 2016; Uranga et al., 2016; Royer et al., 2018; van Duin and Paterson, 2020). Conversely, a longer treatment period puts patients at risk of antibiotic-associated adverse events, such as clostridioides difficile infection and multidrug-resistant (MDR) microorganisms (Vaughn et al., 2019; Wattengel et al., 2019; Du et al., 2021). Antibiotic abuse is becoming more and more serious in China. With its wide application in bacterial pneumonia, the detection rate and infection rate of various drug-resistant bacteria have also increased dramatically (Hu et al., 2018), resulting in a significant increase in the incidence of drug-resistant bacterial pneumonia. Overuse of antibiotics, particularly fluoroquinolones (Møller Gundersen et al., 2019; Gilbert et al., 2020), may increase the risk of MDR bacteria selection and nosocomial pneumonia, though convincing clinical data to support these possibilities are lacking (Tamma et al., 2012). The clinical research of Adrie et al. (2013) showed that the rates of secondary identification rate of MDR bacteria in the monotherapy and dual therapy groups was similar to the incidence of nosocomial pneumonia. This finding should not be construed as supporting evidence that unnecessary antibiotic use is harmless. Widespread abuse of antibiotic does generate more MDR bacteria. Facing the drug-resistant bacterial pneumonia, the choice of antimicrobial drugs based on results of drug sensitivity is very limited, and it is difficult to adequately balance the antimicrobial effect with adverse reactions. At the same time, the development of new antimicrobial drugs is difficult and time consuming (Chellat et al., 2016), making their clinical application very difficult.

For CDBP, the Modern Medical Association refers to the Guidelines for the Diagnosis and Treatment of Chinese Adult Community-Acquired Pneumonia (2016 Edition) and recommends rational use of antibacterial drugs based on the patient’s severity and possible pathogen infection. For example, MRSA is given vancomycin, ESBLs *Klebsiella pneumoniae* is given imipenem/statin sodium (Qu & Cao, 2016) to assist mechanical ventilation, enhance sputum removal, maintain the balance of the internal environment, and encourage the patients to cough, turn, or pat the back to promote sputum excretion, give expectorant and anticonvulsant drugs, and nebulize normal saline when necessary. In addition, immunomodulators such as immunoglobulin, transfer factor, thymidine, etc., can be used in adjuvant therapy to help prevent and treat MOF.

Traditional Chinese medicine (TCM) is a popular complementary treatment and complementary medicine with unique advantages in the treatment of drug-resistant bacterial pneumonia. Modern pharmacological studies have shown that TCM is rich in active ingredients, less prone to drug resistance, and has antibacterial, anti-inflammatory, anti-endotoxic and immune enhancing effects (Han et al., 2016; Cheng et al., 2019). Although Western medicine antibacterial drugs have strong antibacterial effects, they have many side effects, such as allergies, double infection, bacterial resistance, which limit their clinical application. Studies have shown that the antibacterial spectrum of single Chinese medicine is narrow, and the antibacterial effect is difficult to adapt to the needs of complex diseases. Traditional Chinese medicine compound has the characteristics of multi-component, multi-channel and multi-target. It has a wide antibacterial spectrum, and has the advantages of high efficiency, low toxicity and non-resistance. The combination of TCM prescriptions and western drugs not only give full play to the therapeutic characteristics of individualized TCM identification, dose reduction and multi-channel overall regulation, but also can better alleviate clinical symptoms and improve the patient’s regression and prognosis. In recent years, a large number of studies have been conducted to find effective alternative or supplements for the treatment of drug-resistant bacterial pneumonia with TCM or a combination of Chinese and Western medicine.

Yinhuapinggan granules (YHPG) were developed by Shaanxi Dongke Pharmaceutical Company Limited (Shaanxi, China) and approved by the Chinese Food and Drug Administration (CFDA) for CDBP treatment in 2002 (Patent No. ZL031 51188.0; Approval No. Z20184088). YHPG consists of six herbs (Table 1) that clear heat, detoxify the body and promote lung penetration to expel the evil influence. Studies on animal and cellular pharmacodynamic and mechanism of action of YHPG have shown that it has various effects such as antiviral, antibacterial, antipyretic, analgesic, cough suppressant and immune regulator. Toxicological studies did not show any significant toxic side effects. YHPG have significant preventive and curative effects on influenza virus pneumonia in mice, which can be achieved by regulating the secretion of inflammatory factors in the body and reducing the inflammatory response of respiratory organs (Peng et al., 2016a). After treatment, YHPG can significantly reduce the lung index and lung tissue viral load of influenza virus H1N1-infected mice, and alleviate the pathological changes of lung tissue, which can inhibit influenza virus replication and regulate the decrease of immune function in influenza virus mice (Peng et al., 2015). The mechanism of anti-influenza viral action of YHPG may be related to the attenuation of lung injury in influenza virus-infected mice and the regulation of gene and protein expression of key targets of TLRs signaling pathway (Peng et al., 2016b; Du et al., 2017). However, current clinical data on YHPG as CDBP complementary therapy lack high quality and have limited methodology and sample size. Therefore, we designed a double-blind clinical trial to investigate the efficacy and safety of YHPG for the treatment of acquired pneumonia (Wang et al., 2020). The main hypothesis of this research was as follows: in combination with conventional standard therapy, YHPG was superior to placebo in patients with acquired pneumonia.

**TABLE 1:** Components of YHPG (intervention drug).

Scientific name	Chinese Pinyin	Latin scientific name	Parts and form used
*Lonicera japonica* Thunb.	Jin Yin Hua	*Lonicerae japonicae flos*	Dried flower bud
*Ephedra sinica* Stapf.	Ma Huang	*Ephedrae herba*	Dried root and rhizome
*Prunus armeniaca* L. var. ansu Maxim.	Ku Xing Ren	*Armeniacae semen amarum*	Dried mature seeds
*Polygonum cuspidatum* Sieb.et Zucc.	Hu Zhang	*Polygoni cuspidi rhizoma et radix*	Dried root and rhizome
*Pueraria lobata* (Willd.) Ohwi.	Ge Gen	*Puerariae lobatae radix*	Dried root
*Glycyrrhiza uralensis* Fisch.	Gan Cao	*Glycyrrhizae radix et rhizoma*	Dried root and rhizome

## Methods and Design

### Design and Settings

**FIGURE 1:**
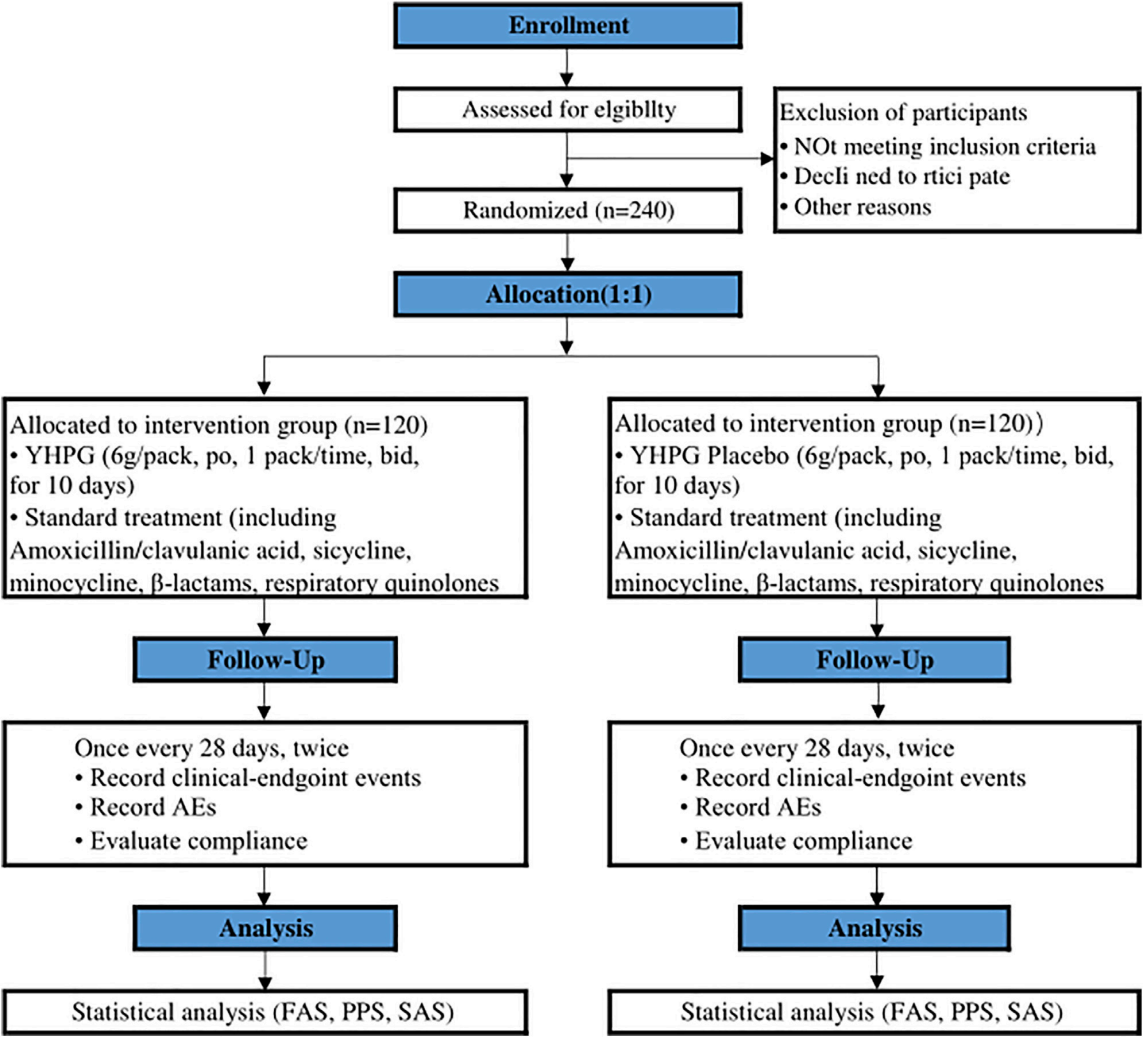
Flowchart of the clinical trial design. The template is from the CONSORT 2010 flowchart. YHPG, Yinhuapinggan; AEs, adverse events; FAS, full analysis set; PPS, per protocol analysis; SAS, safety assessment set.

This research is a prospective, randomized, double-blind, placebo-controlled, superior trial (Wang et al., 2021). The trial will be conducted at Hangzhou First People’s Hospital and will enroll 240 participants. Participants will be randomly assigned to either the YHPG group or the placebo group in a 1:1 ratio after enrolling and obtaining written informed consent. This trial includes a 7-day baseline period, a 10-day intervention period, and a 56-day follow-up period. A flow chart of the research process is shown in Figure 1 investigators will monitor and assess participants at each visit. The design follows the rules of the Interventional Trial Standards Protocol Project Proposal (Chan et al., 2013) and the Consolidated Standards for Reporting Trials (CONSORT) (Schulz et al., 2010).

### Recruitment

The research will be conducted at Hangzhou First People’s Hospital. The recruitment strategy will include advertising in local free newspapers, social media, online publications and posters displayed by participating institutions. Recruitment begins in August 2021 and will be completed over a 2-year period. Patients who agree to participate will be examined and diagnosed by an associate attending physician to confirm their inclusion in the research and will be enrolled in the online allocation system after written informed consent is obtained.

### Study Population

#### Inclusion Criteria

- Voluntary participation, understanding and signing the informed consent form. Be aged 18–85 years, regardless of gender.
- Meet the diagnostic criteria for community-acquired drug-resistant bacterial pneumonia (positive bacterial culture for drug-resistant bacteria in lower respiratory tract specimens).
- Chinese medicine evidence of evil stagnation of the lung and Wei evidence or phlegm-heat congestion of the lung (pneumonia mild to moderate, evidence of Wei-Qi division).
- Satisfy all of the above at the same time.

#### Exclusion Criteria

- Those whose family or patients do not agree or cannot comply with the treatment.
- Patients with lung infections caused by lung tumors, tuberculosis; or other non-infectious pneumonia; combined with severe heart and brain disease.
- Hospital-acquired drug-resistant bacterial pneumonia; those who are allergic to western or Chinese medicine and have already used Chinese medicine to treat patients.
- Multi-drug resistant patients.

### Criteria for Withdrawal, Removal, Dropout and Termination

The investigator’s decision to withdraw the case from the trial refers to a situation in which a subject who has been enrolled in the trial becomes unfit to continue the trial, for example: 1) if the subject’s condition worsens during the course of the research, the subject is allowed to complete the safety examination and withdraw from the trial to receive other effective treatment in order to protect the subject; 2) during the clinical trial, the subject develops certain comorbidities, complications or special physiological changes that make the subject unfit to continue the trial. 3) the use of other treatments or drugs that are prohibited from being used together, which affects the determination of efficacy and safety; 4) the occurrence of adverse events or serious adverse events that make the subject unfit to continue with the trial; 5) the use of less than 80% or more than 120% of the prescribed amounts of drugs; 6) blinding or emergency blinding.

Subjects have the right to withdraw from the trial in the middle of the trial according to the provisions of the informed consent form; or subjects who have not explicitly requested to withdraw from the trial but are no longer receiving the drug and testing and are lost to follow-up are referred to as disenrollment. The reasons for withdrawal or drop-out should be known and recorded whenever possible. For example, if the subject feels that the treatment is not effective, if he/she has difficulty tolerating certain discomforts, if there is something that prevents him/her from continuing with the clinical research, if there are financial factors, or if he/she has not given a reason for dropping out.

When a subject withdraws from the trial, the investigator must complete the reason for withdrawal in the eCRF, contact the subject if possible, complete the assessment items that can be completed, and complete an end-of-treatment follow-up record form, recording the last dosing time if possible. For those discharges due to adverse events that are finally judged to be related to the trial drug after follow-up, this must be recorded in the eCRF and the sponsor notified.

The entire research will be terminated if 1) a serious adverse event related to the trial drug occurs during trial implementation; 2) major deviations or human errors are identified during trial implementation that seriously affect the quality of the trial and make it difficult to achieve the trial objectives; 3) termination is requested by the sponsor with full protection of the rights and safety of the subjects (e.g., funding reasons, administrative reasons, etc.); 4) termination is ordered by the State Food and Drug Administration or the Ethics Committee for one reason or another.

All requirements set out in the research protocol must be strictly enforced. Any intentional or unintentional deviation or violation of the trial protocol and GCP principles can be classified as a deviation from the protocol or violation of the protocol. In the course of monitoring, if a deviation from the protocol is found by the supervisor, a deviation record should be completed by the investigator or supervisor, detailing the time of discovery, the time and process of the event, the cause and the corresponding handling measures, signed by the investigator and communicated to the Ethics Committee and the sponsor. In the data statistics and summary report, the investigator analyses and reports on the impact of protocol deviations or breaches that occur on the final data and conclusions.

When serious protocol breaches occur, an assessment should be made. If necessary, the sponsor may terminate the research early.

### Randomization, Allocation Concealment Mechanism, and Blinding

After obtaining informed consent from all patients, participants are assigned to two random factors in a 1:1 ratio. The randomization list was generated by computer based on randomly arranged blocks from the test statistician. According to the list, the test drug (including placebo) was blind-labeled and packaged by Shaanxi Dongke Pharmaceutical Co., Ltd., for shipment to the trial site; treatments were randomly assigned by removing sequentially numbered treatments from the relevant supply.

Double-blind controlled trials are blinded to the investigator and the subject. The blinding process is carried out by the person in charge of the clinical research unit in conjunction with the sponsor and the statistician, and the test and control drugs are packaged in a uniform manner, while ensuring that there is no difference in appearance between the two groups of drugs, and the numbering of the drug boxes is blinded. The blinding process should be documented and signed by all staff involved in the blinding process. The blinded base must be sealed on the spot after the drug has been dispensed and kept in two separate locations by the institutional staff responsible for the clinical research and the sponsor. Emergency letters should be sent to each center with the trial drug and kept by the person in charge of the institution until the end of the trial.

In case of emergency, the investigator should ask the head of the center for permission to open the emergency blinded letter and notify the clinical research team leader within 24 h after the blind is broken.

### Interventions

Eligible participants will be randomly assigned to either the YHPG group or the placebo group. If YHPG was used prior to randomization, a 2-week drug washout period should be implemented to avoid any potential complications from YHPG. All participants will receive standard treatment.

The YHPG group will receive YHPG (batch no. 20210602, 6 g per packet, manipulated, one packet at a time, 2 times per day). The placebo group will receive YHPG simulant (batch no. 20210602, 6 g per packet, operated, one packet at a time, 2 times per day).

Regarding standard treatment, oral anti-infective drugs with good therapeutic bioavailability such as amoxicillin/clavulanic acid, ciclosporin, minocycline, β-lactams, and respiratory quinolones should be used during the intervention period according to CDBP treatment guidelines (Qu & Cao, 2016). Herbal formulations containing similar ingredients and efficacy to YHPG will not be permitted. Concomitant treatment of comorbidities (e.g., hypertension, diabetes, hyperlipidemia, and other chronic conditions) is permitted during the intervention. Investigators should document concomitant medications faithfully and maintain dose stability during the trial.

With regard to emergency treatment, participants should be treated first in the event of SAE or acute deterioration during treatment and treatment status should be recorded as AE record sheet and combined drug record sheet. If the participant’s condition worsens during treatment and continuation of the trial is not recommended (e.g., local or systemic complications such as PE, ARDS, empyema, lung abscess, phlebitis, sepsis, and metastatic abscess), the trial should be considered for termination and conversion to surgery or other types of clinical therapy. Patients will be categorized as “treatment ineffective” in the analysis.

YHPG and capsule mimics will be supplied by Shaanxi Dongke Pharmaceutical Co. The quality control of YHPG is important. The method of determining the composition of YHPG is based on the general principles of “The Chinese Pharmacopoeia (2020 Edition).” Using gas chromatography (General Principle 0512) (Compiled by the National Pharmacopoeia Commission, 2019), each package of YHPG should contain chlorogenic acid (C_16_H_18_O_9_) ≥ 7.5 mg, lignan (C_21_H_20_O_11_) ≥ 0.25 mg, rhodopsin (C_15_H_10_O_5_) ≥ 1.5 mg, polydatin (C_20_H_22_O_8_) ≥ 0.38 mg and puerarin (C_21_H_20_O_9_) ≥ 6 mg. The main contents of the capsule mimics are corn starch, silica, caramel (liquid) and sunset yellow. We added 2% YHPG powder to the capsule mimic to achieve an odor, color, taste, and texture comparable to YHPG. After treatment, the packages will be returned to the researchers.

## Outcomes

### Primary and Secondary Outcomes

Details of the items to be measured and the time window for data collection are shown in Table 2. The primary outcome was the cure rate of pneumonia (at different time periods). The efficacy was determined by referring to the “Guidelines for Clinical Research on New Chinese Medicines” evaluation method (Editor-in-Chief Zheng Xiaoyu, 2002), and the nimodipine method was used.

n (efficacy index) = (pre-treatment score - post-treatment score)/pre-treatment score × 100%

**TABLE 2:** Research schedule.

Research phase time	Baseline period	Intervention period		Follow-up	
	Visit 1	Visit 2	Visit 3	Visit 4	Visit 5
	−7 to 0 days	5 days	10 days	28 days	56 days
Data collection at baseline	×				
Informed consent	×				
Inclusion/exclusion criteria	×				
Demographic data	×				
Obtain the central random number	×				
Previous history, medical history, and allergies	×				
Comorbidities and co-medications	×				
Safety evaluation					
Vital signs	×	×	×		
Physical examination	×	×	×		
Blood routine	×		×		
Urine routine	×		×		
Blood biochemistry	×		×		
ECG	×		×		
Urine pregnancy test	×		×		
Efficiency evaluation					
Pneumonia cure rate		×	×		
Diagnosis of severe pneumonia	×	×	×		
Laboratory tests	×	×	×		
APACHEII score	×		×		
SMART-COP score	×		×		
Lower respiratory tract bacterial clearance		×	×		
LC clearance rate		×	×		
Clinical-endgoint events		×	×	×	×
Other work					
Dispense drug	×				
Recovery and record of research drug			×		
Record AEs		×	×	×	×
Complications due to medications		×	×		
Evaluate compliance		×	×	×	×

ECG, Electrocardiogram Laboratory tests, C-reactive protein, Lactic acid, Procalcitonin, Oxygenation index; APACHEII score, Acute Physiology, Age and Chronic Health Evaluation; LC, lactic acid; AEs, adverse events.

Markedly effective: clinical symptoms and signs improved significantly, 70% ≤ *n* < 100%; Effective: clinical symptoms and signs improved, 30% ≤ *n* < 70%; Ineffective: clinical symptoms and signs did not improve significantly, or even worsened, *n* < 30%.

Pneumonia cure rate = (number of effective cases + number of effective cases)/total number of cases × 100%

Secondary outcomes were 1) rate of severe pneumonia; 2) criticality score (SMART-COP score) (Charles et al., 2008); 3) acute physiological and chronic health assessment system (APACHEII score) (Knaus et al., 1985); 4) temperature, lower respiratory bacterial clearance, LC clearance rate, CRP, Procalcitonin (PCT), and oxygenation index (PaO2/FiO2); 5) clinical endpoint event incidence (acute respiratory failure, septic shock, acute heart failure, acute coronary syndrome, and all-cause death).

### Safety Evaluation Standard

Safety evaluation standard include AEs, SAEs, concomitant medication, changes in clinical laboratory results (e.g., blood routine, blood biochemistry, urine routine, etc.), clinical symptoms, results of vital sign measurements such as temperature, heart rate, respiratory rate, blood pressure, 12-lead ECG and physical examination.

### System Biological Index

Fifty subjects in each group will be randomly selected for metabolic, proteomics, and transcriptional examinations to explore the biomarkers of YHPG for the treatment of CBAP (Wang et al., 2020).

Regarding the collection of blood samples, participants will fast for 10 h before sampling at the start of the research (day 0) and after treatment (day 10). 5 ml of blood will then be drawn and centrifuged and the serum stored in EP tubes at −80°C. Regarding the collection of urine samples, participants will fast for 10 h (and be allowed to drink water) before taking the medication. They will be fasted and water consumption will be prohibited up to 2 h before urine collection 1 h after taking the drug. After taking the drug, water will be rationed to 200 ml/h for 2–8 h and low-fat meals may be consumed for 4 h after taking the drug. Urine samples will be collected at baseline and after treatment (10 days) and stored in EP tubes at −80°C.

### Collection and Management of Data

The EDC (eCRF) system will used for this research. The investigator will complete the electronic case report form completely and accurately. Only one clinical research subject’s data information will be recorded on each case report form.

After data entry into the eCRF at the research center, quality control staff should check the consistency of the eCRF data with the original records to ensure that the data are accurately completed into the eCRF. The supervisor verifies 100% that the trial data entry in the eCRF system is complete, accurate and consistent with the original medical record information. Data items that are in doubt, or inconsistent with the original medical record information, are promptly challenged. Data entry clerks and investigators were urged to respond to queries and to verify and correct inconsistent data.

Data management staff use logical verification to verify the quality of data entry, and the results of queries are sent to the investigator in the form of a challenge, which is verified and corrected by the investigator. Quality control staff verify data management files and database data.

The research center quality assurance staff carry out random checks on data transfer files and statistical report data to ensure that the data are accurate. The sponsor conducts audits in different areas of the above clinical trial process, sample testing process, data, reporting and calculation process as needed, taking into account the progress of the trial and the results of the quality control staff/monitors’ verification.

Throughout the trial, all data obtained during the clinical research will be handled appropriately to ensure the rights and privacy of the subjects participating in the clinical research and the investigators will maintain the confidentiality of the data for a period of 5 years after the completion of the trial.

### Sample Size

The formula for calculating the sample size was based on superior clinical trial sample size estimates (Wan et al., 2017a). According to the need of the research, the clinical research part was focused on preliminary exploration, and a small sample size was selected to conduct the research on the early effects of herbal interventions in pneumonia (whether the interventions turned into severe pneumonia) with reference to previous relevant clinical studies on exogenous fever in our project group (unpublished data).

Refer to the clinical research literature of our team for treating pneumonia (Wan et al., 2017b), the healing rate was 85%–89% in the treatment group and the placebo group was 69%–74%. Given an error rate of *α* = 0.025 for the treatment group and β = 0.2 for the control group. Taking into account the dropout rate of 10%, it is calculated that 216 patients should be allocated to the treatment group and the placebo group in a 1:1 ratio. In conjunction with the need for clinical laboratory sample analysis, a total of 240 cases are therefore proposed to be enrolled, 120 in each group.

### Trial Completion

The trial will end after 240 patients have been randomized and all patients have completed 56 days of treatment and follow-up.

### Statistical Analyses

EPI 3.0 software was used for data management; SAS software package was used for statistical analysis. All statistical tests were performed using a two-sided test, and a *p*-value less than or equal to 0.05 was considered statistically significant for the difference tested. Different subgroups of drug-resistant bacteria were also analyzed according to pathogen detection.

Measures for each visit in the different treatment groups will be statistically described using the mean ± standard deviation. Comparisons will be made with the basal values of the screening period and paired t-tests will be used to compare pre- and post-group differences. Changes in the two groups before and after treatment for the primary indicators are compared using analysis of covariance (COANOVA). Comparisons between groups for measures of secondary indicators were made using paired t-tests or rank sum tests.

The statistical description of the count data for each visit in the different treatment groups was done using frequency (composition ratio). Changes before and after treatment in the two groups were tested using the *χ* ^2^ test or the exact probability test. Grade information was compared between groups using the rank sum test. Table 3 displays the analysis method for a specific result.

**TABLE 3:** Outcomes and methods of analyses.

Outcome/variable	Hypothesis	Measures	Methods of analyses
Baseline balance test		Quantitative outcomes (age, temperature, heart rate, respiratory rate and blood pressure)	t-test/Wilcoxon rank-sum test
		Qualitative outcomes (sex, marriage and previous treatment)	Chi-squared test/Fisher’s exact test/rank-sum test
Adherence at post-lntervention		Percent and cases Of adherence <80% and ≥80%	Chi-squared test/Fisher’s exact test
Concomitant treatments		Percent and cases of concomitant treatments	Chi-squared test/Fisher’s exact test
Primary outcome			
Pneumonia cure rate	Improvement occurred		t-test/Wilcoxon rank-sum test Covariance analysis
Secondary outcomes			
Severe pneumonia rate	Improvement occurred		t-test/Wilcoxon rank-sum test
Laboratory tests	Improvement occurred		t-test/Wilcoxon rank-sum test
APACHEII score	Improvement occurred		t-test/Wilcoxon rank-sum test
SMART-COP score			t-test/Wilcoxon rank-sum test
Lower respiratory tract bacterial			t-test/Wilcoxon rank-sum test
clearance rate			
LC clearance rate			t-test/Wilcoxon rank-sum test
Safety outcomes			
AEs, SAE		Percent and cases Of AEs and SAEs	Chi-squared test/Fisher’s exact test
Vital signs		Change value relative to baseline	t-test/Wilcoxon rank-sum test

Laboratory tests, C-reactive protein, Lactic acid, Procalcitonin, Oxygenation index; APACHEII score, Acute Physiology, Age and Chronic Health Evaluation; LC, lactic acid; AEs, adverse events; SAE, serious adverse events.

### Adverse Events

All clinical events and clinically significant laboratory adverse reactions will be handled in accordance with the harmonized guidelines detailed in the Common Adverse Events Evaluation Criteria NCI CTCAE Version 4.03 and Emergency Response Reference Plan. Clinical events and clinically significant laboratory test abnormalities will be graded in accordance with NCI CTCAE 4.03. Treatment-induced adverse reactions will be documented by the investigator and brought to the attention of the sponsor’s medical monitor, who will discuss with the investigator and determine appropriate action steps. All subjects experiencing AEs, whether or not they are considered treatment-related, must be monitored regularly (if feasible) until symptoms subside, any abnormal laboratory values return to normal or to baseline levels or until they are considered irreversible, or until the observed changes can be appropriately interpreted. Abnormalities in laboratory tests of clinical significance at level 3 or 4 should be confirmed by repeat testing, if practicable, preferably within 3 working days of receipt of the initial test result.

### Quality Control of the Intervention

In order to further ensure the quality of clinical trials, a multi-center trial coordination committee is established, with the general director of the clinical research and acting as the coordinator of the research among the centers of the clinical trial, and the research director of each participating research unit and the director of the sponsor as members of the coordination committee, which is responsible for the implementation of the entire trial and the research and resolution of trial-related issues.

Supervisors are appointed by the sponsor to ensure that the rights and interests of subjects in clinical trials are protected, that data in trial records and reports are accurate and complete, to oversee the implementation of clinical trial protocols, drug clinical trial management norms and relevant regulations, and to conduct regular on-site supervision visits to each center. The investigator shall agree that the clinical trials conducted are subject to inspection and audit by the State Food and Drug Administration, or visual inspection or audit by the sponsor or CRO.

Clinical supervisors have the right to propose amendments to eCRF forms that do not comply with the protocol during the monitoring process. Investigators participating in clinical trials are responsible for making corrections to eCRF forms that are not standardized, such as input errors, but must follow the authenticity of the information.

Through pre-clinical trial training, researchers should be fully aware of and understand the clinical trial protocol and the specific content of its indicators. For the objective indicators specified, they should be checked at the time points and methods specified in the protocol, and attention should be paid to observing adverse reactions or unanticipated toxic effects and following up on them.

### Trial Status

This is an ongoing trial. The first participant was enrolled in 16 August 2021; As of 31 December 2021, a total of 78 patients have been enrolled. Recruitment is scheduled to be completed in December 2022 and analysis will be completed by April 2023. The trial will end after the last follow-up visit of the last random participant.

## Discussion

CDBP is a common and prevalent clinical condition and one of the most common infectious diseases, in which the use of antimicrobial drugs is most important. And in the face of drug-resistant bacterial pneumonia arising from the proliferation of antimicrobial drugs, the choice of antimicrobial drugs based on drug sensitivity results is very limited, and it is difficult to make an adequate balance between antimicrobial effects and adverse effects (Møller Gundersen et al., 2019; Spellberg and Rice, 2019). Meanwhile, novel antimicrobial drugs are difficult to develop and have long lead times (Chellat et al., 2016), making their clinical treatment very difficult. A growing body of evidence suggests that the combination of TCM and Western medicine may be the best approach to achieve greater therapeutic efficacy in patients with CDBP.

YHPG is a widely used herbal formulation for the treatment of acquired pneumonia in China. Pharmacological results have shown that YHPG is effective in a variety of mechanistic pathways for the treatment of CDBP, such as anti-influenza virus (Peng et al., 2016b; Du et al., 2017), preventing and controlling influenza virus pneumonia, reducing the viral load of influenza virus in lung tissue, attenuating lung histopathological changes, inhibiting influenza virus replication and regulating immune function (Emily et al., 2011). However, whether YHPG is effective in CDBP patients’ needs to be confirmed by a large sample, randomized controlled clinical trial. Therefore, we designed a randomized, double-blind, placebo-controlled clinical trial in the hope of validating the efficacy and safety of CDBP for the treatment of CDBP (Wang et al., 2021). The results of the research may provide a strategy for the combined treatment of Chinese and Western medicine in CDBP.

We chose the efficacy index of pneumonia (different time periods) (efficacy determination was based on the evaluation method of the “Guidelines for Clinical Research on New Chinese Medicines”, using the nimodipine method) to assess the cure of CDBP patients (Editor-in-Chief Zheng Xiaoyu, 2002). The efficacy index for pneumonia (different time periods) is a convenient and intuitive criterion that does not require special equipment or advanced training for physicians and is well tolerated by patients. The degree of patient recovery is then assessed by the criticality score (SMART-COP score) (Charles et al., 2008), and the Acute Physiological and Chronic Health Assessment System (APACHEII score) (Knaus et al., 1985). In the meantime, patient temperature, lower respiratory bacterial clearance, LC clearance rate, CRP, PCT, and oxygenation index (PaO2/FiO2) together form part of the efficacy index.

In addition, we applied metabolic and proteomics analyses to explore therapeutic biomarkers of YHPG for CDBP and to improve CDBP treatment by providing an objective basis for precise treatment (Wang et al., 2020).

However, several limitations need to be considered. Firstly, the YHPG used in this trial is for the treatment of early and mid-stage CDBP, so these findings may not be applicable to severe CDBP. secondly, the AEs will only be recorded and treated during the 10-day intervention period and 56-day follow-up period, which is a relatively short period, but the short-term results could encourage further prospective studies with different treatment regimens and longer follow-up times. Finally, this trial will be conducted at Hangzhou First People’s Hospital, and the results of treatment effects and individual differences in YHPG in other areas are not available. More efforts should be made to optimize these shortcomings and answer these questions in future research.

## Data Availability

The original contributions presented in the study are included in the article/supplementary material, further inquiries can be directed to the corresponding authors.
