# Corrigendum: Clinical efficacy protocol of yinhuapinggan granules: A randomized, double-blind, parallel, and controlled clinical trial program for the intervention of community-acquired drug-resistant bacterial pneumonia as a complementary therapy

**DOI:** 10.3389/fphar.2022.986640

**Published:** 2022-08-16

**Authors:** Jiaoli Wang, Haoran Hu, Haixia Du, Man Luo, Yilan Cao, Jiaping Xu, Tianhang Chen, Yilei Guo, Qixiang Li, Wen Chen, Yifei Zhang, Jin Han, Haitong Wan

**Affiliations:** ^1^ Zhejiang Chinese Medical University, Hangzhou, China; ^2^ Department of Respiratory Medicine, Hangzhou First People’s Hospital, Hangzhou, China; ^3^ College of Basic Medical Science, Zhejiang Chinese Medical University, Hangzhou, China; ^4^ College of Life Science, Zhejiang Chinese Medical University, Hangzhou, China; ^5^ Dongzhimen Hospital, Beijing University of Chinese Medicine, Beijing, China

**Keywords:** Yinhuapinggan (YHPG), traditional Chinese medicine (TCM), community-acquired drug-resistant bacterial pneumonia (CDBP), multidrug resistance, clinical trial, clinical efficacy

In the published article, there was an error in affiliations **1**, **2**, **3** and **4**.

Instead of “Jiaoli Wang^1^, Haoran Hu^2^, Haixia Du^3^, Man Luo^1^, Yilan Cao^2^, Jiaping Xu^1^, Tianhang Chen^3^, Yilei Guo^2^, Qixiang Li^2^, Wen Chen^1^, Yifei Zhang^5^, Jin Han^3^* and Haitong Wan^4^*

^1^Department of Respiratory Medicine, Hangzhou First People’s Hospital, Hangzhou, China, ^2^College of Basic Medical Science, Zhejiang Chinese Medical University, Hangzhou, China, ^3^College of Life Science, Zhejiang Chinese Medical University, Hangzhou, China, ^4^Zhejiang Chinese Medical University, Hangzhou, China, ^5^Dongzhimen Hospital, Beijing University of Chinese Medicine, Beijing, China.”

It should be “Jiaoli Wang^1,2†^, Haoran Hu^3†^, Haixia Du^4^, Man Luo^2^, Yilan Cao^3^, Jiaping Xu^3^, Tianhang Chen^4^, Yilei Guo^3^, Qixiang Li^3^, Wen Chen^3^, Yifei Zhang^5^, Jin Han^3^* and Haitong Wan^1,4^*

^1^Zhejiang Chinese Medical University, Hangzhou, China, ^2^Department of Respiratory Medicine, Hangzhou First People’s Hospital, Hangzhou, China, ^3^College of Basic Medical Science, Zhejiang Chinese Medical University, Hangzhou, China, ^4^College of Life Science, Zhejiang Chinese Medical University, Hangzhou, China, ^5^Dongzhimen Hospital, Beijing University of Chinese Medicine, Beijing, China

^†^These authors have contributed equally to this work.”

The authors apologize for this error and state that this does not change the scientific conclusions of the article in any way. The original article has been updated.

